# The activated DNA double-strand break repair pathway in cumulus cells from aging patients may be used as a convincing predictor of poor outcomes after *in vitro* fertilization-embryo transfer treatment

**DOI:** 10.1371/journal.pone.0204524

**Published:** 2018-09-20

**Authors:** Xu-lei Sun, Hao Jiang, Dong-xu Han, Yao Fu, Jian-bo Liu, Yan Gao, Shu-min Hu, Bao Yuan, Jia-bao Zhang

**Affiliations:** 1 Department of Laboratory Animals, College of Animal Sciences, Jilin University, Changchun, Jilin, P.R. China; 2 Shenyang Jiuzhou Family Hospital, Shenyang, Liaoning, P.R. China; Universite Laval, CANADA

## Abstract

Women with advanced maternal age exhibit low anti-Müllerian hormone (AMH) levels and an altered follicular environment, which is associated with poor oocyte quality and embryonic developmental potential. However, the underlying mechanism is poorly understood. The present study aimed to assesswhether aging patients exhibit an activated DNA double-strandbreak (DSB) repair pathway in cumulus cells and thus, an association with poor outcomes after in vitro fertilization-embryo transfer (IVF-ET) treatment. Cumulus cells from young (≤29 y) and aging (≥37 y) human female patients were collected after oocyte retrieval. Our results indicated that aging patients showed a higher rate of γ-H2AX-positive cells than in young patients (24.33±4.55 vs.12.40±2.31, P<0.05). We also found that the mRNA expression levels of BRCA1, ATM, MRE11 and RAD51 were significantly elevated in aging cumulus cells. Accordingly, significantly increased protein levels of phospho-H2AX, BRCA1, ATM, MRE11 and RAD51 could be observed in aging cumulus cells. Moreover, aging cumulus cells showed a more frequent occurrence of early apoptosis than young cumulus cells. This study found that increases in DSBs and the activation of the repair pathway are potential indicators that may be used to predictoutcomes after IVF-ET treatment.

## Introduction

Cumulus cells, which are layers of specialized granulosa cells encircling oocytes, are thought to be closely related to the growth and meiotic maturation of oocytes, not only through supplying nutrients or signaling molecules to oocytes via gap junctions [1,2] but also via the protection of oocytes from adverse environments or factors [3]. Notably, increasing evidence has demonstrated that apoptosis in cumulus cells plays a mediating role in impairing oocyte developmental potential [4–6]. Thus, many studies have attempted to identify efficient and convincing biological markers in cumulus cells to predict the quality of oocytes and their developmental competence [7–9]. For example, the expression of gremlin 1 (GREM1) and hyaluronan synthase 2 (HAS2) is correlated with the developmental ability of oocytes [8]. Telomere length in cumulus cells is predictive of the competence of oocytes and embryos but may not be sufficiently discriminating to be clinically useful because of the limited number of cumulus cells [10]. Loss of cumulus gene expression has been observed in abnormal or dysfunctional cumulus cells and, thus, is thought to be associated with poor performance of subsequent embryo development [9].

During the process of apoptosis, the most deleterious type of DNA damage may be DNA DSBs, which result in chromosomal instability and failed rearrangements. If DSBs are not promptly and accurately repaired, they may cause a wide range of genome aberrations, resulting in cell cycle arrest or apoptosis [11,12]. Therefore, DSBs are thought to be a determining trigger of apoptosis [13]. In response to DSBs, cells must initiate a cascade of biological pathways to promote the repair of DNA damage to survive and restore genome integrity, thereby preventing the transmission of false genetic information and protecting genetic integrity [14].

Previous studies on DSB repair pathways have focused on the targeted treatment of cancers. One early study showed that the ovarian reserve function decreases significantly in women who survive cancer chemotherapy. Many cancer patients treated via chemotherapy display a decrease in or loss of fertility, suggesting that chemotherapeutic drugs may damage ovarian function and oocyte developmental potential [15]. Further research using in vitro-cultured human ovarian tissue and in vivo experiments in mice showed that chemotherapeutic drugs induced notable DSBs in humans and rodent primary follicles, oocytes and granulosa cells, which were associated with the activation of ataxia-telangiectasia mutated (ATM) [16].

Breast cancer susceptibility gene1 (BRCA1) is a crucial member of the ATM-mediated DSB repair gene family, and mutation of this gene is associated with a high risk of breast, ovarian, and other cancers [17–19]. Previous studies have indicated that women who carry a mutated *BRCA1* gene in their germline show a lower ovarian stimulation response and even experience earlier menopause. These findings support a role of DNA DSB repair in the maintenance of human ovarian reserves [17–19]. In addition, these studies also identified DSBs in oocytes, resulting in a decline of reproductive capacity.

With advancing maternal age, the reproductive capacity decreases gradually, which is observed as a decrease in ovarian reserves and a significantly increased rate of oocyte loss [20]. In many cell types, including oocytes and granulosa cells, DNA damage increases with aging. γ-H2AX is a marker of DSBs. After DSB recognition, the histone H2AX is phosphorylated on Ser139, resulting in γ-H2AX foci at the sites of DNA damage, which can be employed as a measure of the extent of DSBs and further recruit DNA repair proteins [21].

Results from rhesus monkeys indicate that increases in DSBs and a deficient repair pathway in granulosa cells, characterized by an increase in γ-H2AX foci and decrease in BRCA1 foci are related to ovarian aging [22]. Studies in human and rodent models also showed that DSBs increase in oocytes with maternal age, and the corresponding expression of some DSB repair genes (such as BRCA1) decreases in oocytes [23,24]. These findings suggest that an increase in DSBs and a reduced repair capacity in both oocytes and granulosa cells may play a central role in triggering apoptosis and, thus, accelerating ovarian aging [24,25].

These observations led us to hypothesize that an increase in DSBs and, thus, activation of the repair pathway may be used as convincing indicators to predict the outcome of IVF cycles in patients showing risk factors such as maternal aging. Here, using cumulus cells from aging patients, we demonstrated that aging cumulus cells are characterized by an activated DSB repair pathway as well as changes in the expression of related genes. These results are consistent with the translocation of phosphatidylserine (PS), which is a hallmark of early apoptosis. Finally, these observations are associated with a significantly lower developmental potential as well as the poor outcomes after IVF-ET cycles in our tested aging populations. Our findings also offer new insight for further understanding the role of cumulus cells in mediating the poor reproductive performance of aging patients.

## Materials and methods

### Research objective and grouping

Ovarian stimulation was performed via application of a long-term luteal gonadotropin releasing hormone (GnRH) agonist or a GnRH antagonist based on patient characteristics. Ovulation was induced with human chorionic gonadotropin (hCG) to trigger oocyte maturation. Oocyte retrieval was performed by transvaginal ultrasound 36 h after hCG administration. Since no significant differences between the cumulus cells of MII oocytes exist at the transcriptome level in GnRH agonist and antagonist protocols, the cell data were pooled[26].

We collected human cumulus cells from young (≤29 y) and aging (≥37 y) female patients after oocyte retrieval. The inclusion criteria were as follows: no history of operation in the bilateral ovaries, no other chronic or endocrine diseases, and a BMI<28 kg/m^2^. The exclusion criteria were as follows: hyperandrogenemia, polycystic ovarian syndrome (PCOS) and endometriosis. The young group did not include poor ovarian responders, and the aging group did not include the natural cycle. Fertilization and developmental parameters determined in the laboratory as well as clinical outcomes from April 2016 to June 2017 at Shenyang Jiuzhou Family Hospital were recorded, and retrospective analyses were performed.

The project was revised and approved by the Reproductive Medicine Ethics Committee of Shenyang Jiuzhou Family Hospital (project number 20060105, approval date January 5, 2016).

### Cumulus cell isolation and collection

A portion of the cumulus mass that had expanded well and surrounded the oocyte was removed soon after oocyte retrieval using two 1 ml syringes, avoiding blood cells as much as possible. Cumulus cells used for flow cytometry were pretreated with 80 IU hyaluronidase (Sage In-Vitro Fertilization, USA) in the cell suspension, placed in a new 1.5 ml centrifuge tube, washed with ART-1023 (Sage In-Vitro Fertilization, USA) and centrifuged (500 g, 8 min) two times at room temperature. The cell pellets for RNA and protein extraction werewashed two times with ART 1023 followed by centrifugation (500 g, 8 min) with as little fluid as possible and stored at -80°C.

### Measurement of apoptosis and γ-H2AX via flow cytometry

Cumulus cells were stained to detect apoptosis with FITC annexin V/PI (FITC Annexin V Apoptosis Detection kit, BD PharmingenTM, USA) γ-H2AX-positive cells were detected with an anti-H2AXS139P-Alexa Fluor 488-conjugated antibody (Cell Signal Technology, US). Then, 1 x 10^4^ cells of each group were assayed via flow cytometry (FACSCanto II; BD Biosciences, USA).

### Real-time quantitative PCR (RT-qPCR) analysis

The extraction of total RNA from cumulus cells was performed using the TRIpure Isolation Reagent (Roche, Germany) and quantified in a NanoDrop 2000 spectrophotometer (Thermo, USA). cDNA synthesis was performed using 1 μg of RNA and a fast reverse transcription kit according to the manufacturer’s instructions (Tiangen Biotech, China).

The expression levels of DSB repair genes in cumulus cells were detected using a SYBR Green quantitative PCR protocol with GAPDH as an internal control gene. RT-qPCR was carried out in triplicate in a 20 μl reaction volume using SuperReal PreMix Plus (TIANGEN, China), with 8 μl of sterile-deionized water, 1 μl of cDNA and 0.5 μl of each of the upstream and downstream PCR primers (10 μM), in a Mastercycler1 ep realplex machine (Eppendorf, Germany). For each sample, the threshold cycle (CT) was calculated, and the fold-increase in the experimental sample relative to the control was analyzed using the 2-ΔΔCt method. The primer sequences are shown in Table 1.

**Table 1:** Primer sequences used for RT-qPCR in human samples.

Gene name	Primer sequence 5’ to 3’
BRCA1	F-AGCTGTGTGGTGCTTCTGTGGT
	R-TGGCTGCACAACCACAATTGGG
RAD51	F-TTGGGACTACAGGTGGAATTG
	R-TCCAGGACATCACTGCCAGAG
ATM	F-TGCTCAGTGTTGGTGGACAGGT
	R-TCCATCCTGGGAAAAGTCGGCT
MRE11	F-AATAGCGCAGTGTCCTGGATGC
	R-CAGAAGCACCACCTTGTGGCAA
GAPDH	F-AGGGCTGCTTTTAACTCTGGT
	R-CCCCACTTGATTTTGGAGGGA

### Western blot analysis

hCCs were lysed in lysis buffer (Beyotime Biotechnology, China) containing 1 mM phenylmethylsulfonyl fluoride (PMSF; Beyotime, China). Total protein was then extracted, and the protein concentration was assessed using a BCA protein assay kit (Beyotime Biotechnology, China). After denaturation at 95°C for 5 min, a 50 μg protein sample was subjected to 12% sodium dodecyl sulfate-polyacrylamide gel electrophoresis (SDS/PAGE) and then transferred to a PDVF membrane (0.22 mM, Millipore, Bedford, MA, USA). The blots were incubated overnight with the primary antibodies after blocking for 2 h, after which they were washed three times with Tris-buffered saline containing 1% Tween-20 (Sigma, US), and then incubated with the corresponding secondary antibodies for 1 h at room temperature. Chemiluminescence was detected on X-ray film using an Immobilon Western Chemiluminescent HRP Substrate kit (Millipore, Billerica). Gels were analyzed with Image J software to detect protein levels of phosphorylated H2AX and DSB repair genes in aging and young cumulus cells.

### Statistics

All experiments were repeated at least three times or more different repetitions. SPSS 22.0 analysis software was used for the statistical analyses, and the differences between groups were calculated via one-way ANOVA or using the Chi-square test. The data are presented as the means ± standard deviations (x ± sd). *P < 0.05 was considered to indicate a significant difference, while **P < 0.01 represented a highly significant difference.

## Results

### Basic characteristics and hormone levels of aging and young patients

There were 338 cycles in the group of aging patients (average age, 41.63±3.30) and 322 cycles in the group of young patients (average age, 26.20±2.28). Our data indicated that the aging patients exhibited a greater number of infertile years than the young patients (5.74±5.30 vs. 3.35±2.06, P<0.01). Compared with the young patients, the aging patients displayed lower levels of AMH (1.03±0.99 vs. 5.22±3.30, P<0.01) and LH (3.70±1.97 vs. 4.80±3.07, P<0.01), but higher levels of FSH (8.34±3.75 vs. 5.82±1.76, P<0.01) and E_2_ (52.58±38.07 vs. 46.80±19.32, P<0.05) (Table 2).

**Table 2:** Basic characteristics and hormone levels of the young and aging groups.

Variable	Young	Aging
No. of cycles	322	338
Mean age of female patients(year)	26.20±2.28**	41.63±3.30
Infertility years(year)	3.35±2.06**	5.74±5.30
BMI (kg/m^2^)	24.08±4.71	23.80±3.41
Serum AMH level(ng/ml)	5.22±3.30**	1.03±0.99
FSH (mIU/ml)	5.82±1.76**	8.34±3.75
LH (mIU/ml)	4.80±3.07**	3.70±1.97
E_2_ (pg/ml)	46.80±19.32*	52.58±38.07

The data represent the mean ± SD. Significant difference

*P<0.05

**P<0.01.

BMI, body mass index; FSH, follicle-stimulating hormone; LH, luteotropic hormone; AMH, anti-Müllerian hormone; E_2_, 17b-estradiol.

As shown in Table 3, throughout the stimulation cycle, the aging group exhibited fewer gonadotropin (Gn) days (8.94±2.62 vs 10.27±1.65, P<0.01) and much higher doses of Gn (2598.52±951.55 vs 2339.66±722.18, P<0.01) than the young patients. On the hCG trigger day, the aging patients displayed lower E_2_ levels (1438.63±1277.35 vs 3316.46±2047.96, P<0.01), whereas both LH and P exhibited higher levels in aging patients than in young patients (3.87±3.79 vs 1.03±0.98, P<0.01), (1.87±1.86 vs 1.03±0.55, P<0.01).

**Table 3:** Comparison of Gn use and hormone levels on the hCG injection day between the young and aging groups.

Variable	Young	Aging
Gn duration (days)	10.27±1.65**	8.94±2.62
Total Gn received (IU)	2339.66±722.18**	2598.52±951.55
LH level on hCG injection day (mIU/ml)	1.03±0.98**	3.87±3.79
E_2_ level on hCG injection day (pg/ml)	3316.46±2047.96**	1438.63±1277.35
P level on hCG injection day (ng/ml)	1.03±0.55**	1.87±1.86

The data represent the mean ± SD. Significant difference

*P<0.05

**P<0.01.

Gn, gonadotropins; hCG, human chorionic gonadotropin; P, progesterone.

### Decreased embryonic developmental competence in aging patients

The fertilization rate, development rate, embryo quality and clinical outcomes of the young and aging patients were compared in the subgroups receiving either IVF (Table 4) or ICSI (Table 5) treatment. Within an IVF or ICSI cycle, the aging patients displayed a similar rate of good-quality embryos (D3) to the young patients. However, the blastocyst rate of the aging patients was significantly lower than that of the young patients (IVF: 51.82% vs. 69.50%, P<0.01) (ICSI: 50.79% vs. 60.77%, P<0.05). The aging patients also subsequently showed a lower implantation rate (IVF: 18.64% vs. 53.10%, P<0.01) (ICSI: 25.49% vs. 53.57%, P<0.01) and clinical pregnancy rate (37.78% vs. 70.67%, P<0.01) (ICSI: 39.53% vs. 82.14%, P<0.01) as well as a higher early abortion rate (IVF: 29.41% *vs*. 5.66%, *P*<0.05).

**Table 4:** Comparisons of development rates and clinical outcomes between aging and young patients during IVF cycles.

Variable	Young	Aging
No. of IVF cycles	234	124
Total No. of oocytes	3965	1061
Mean No. of oocytes	16.94±6.32**	8.56±5.80
Fertilization rate (%)	77.76(3083/3965)	80.58(855/1061)
Normal fertilization rate (%)	59.45(2357/3965)	63.34(672/1061)
Cleavage rate (%)	97.99(3021/3083)	97.66(835/855)
Normal Cleavage rate (%)	97.84(2306/2357)	98.07(659/672)
Good-quality embryo rate (%)(D3)	53.84(1269/2357)	52.23(351/672)
Blastocyst formation rate (%)	69.50(1351/1944) **	51.82(157/303)
Mean No. of embryos transferred	1.93±0.30	2.62±0.53
Clinical pregnancy rate (%)	70.67(53/75) **	37.78(17/45)
Implantation rate (%)	53.10(77/145) **	18.64(26/102)
Early abortion rate (%)	5.66(3/53) *	29.41(5/17)

Significant difference

*P<0.05

**P<0.01.

Early abortion, occurring abortion before the 12th week.

**Table 5:** Comparisons of development rates and clinical outcomes between aging and young patients during ICSI cycles.

Variable	Young	Aging
No. of ICSI cycles	88	214
Total No. of oocytes	1490	936
Mean No. of oocytes	16.93±6.78**	4.37±3.86
Total No. of MII oocytes	1262	801
Mean No. of MII oocytes	14.34±6.16**	3.74±3.24
Fertilization rate (%)	87.72(1107/1262)	85.77(686/801)
Normal fertilization rate (%)	79.00(997/1262)	77.40(620/801)
Cleavage rate (%)	98.28(1088/1107)	98.25(675/687)
Normal Cleavage rate (%)	98.50(982/997)	98.22(609/620)
Good-quality embryo rate (%)(D3)	49.65(495/997)	50.48(313/620)
Blastocyst formation rate (%)	60.77(381/627)*	50.79(64/126)
Mean No. of embryos transferred	2.00±0.00	2.30±0.71
Clinical pregnancy rate (%)	82.14(23/28)**	39.53(17/43)
Implantation rate (%)	53.57(30/56)**	25.49(26/102)
Early abortion rate (%)	0(0/23)	5.88(1/17)

Significant difference

*P<0.05

**P<0.01.

### Increased DNA DSB rate in aging human cumulus cells

**Fig 1:**
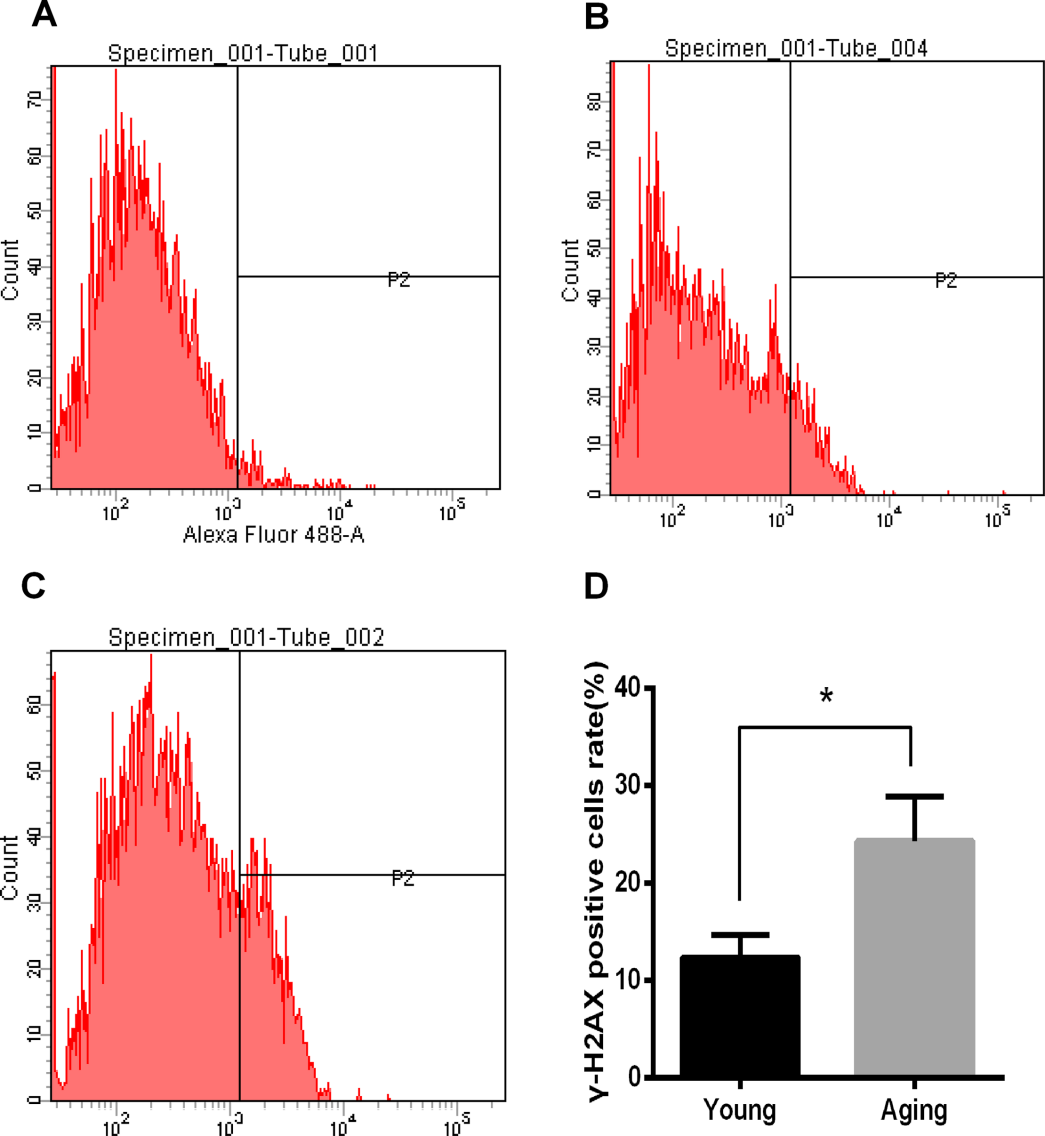
Analysis of DSBs in aging and young cumulus cells. (A, Blank control; B, Young; C, Aging) Representative images of raw flow cytometric data obtained using an anti-H2AXS139P-Alexa Fluor 488-conjugated antibody. (D) Average percentages of γ-H2AX-positive cells in young and aging cumulus cells. The results are reported as the mean ± SD from *n* = 3 independent experiments (significant difference, *P<0.05).

To test our hypothesis, DNA DSBs were assessed via flow cytometric detection of the specific marker γ-H2AX using an anti-H2AXS139P-Alexa Fluor 488-conjugated antibody. We found that the percentage of γ-H2AX-positive cells increased significantly in aging cumulus cells (24.33±4.55 vs.12.40±2.31, P<0.05) (Fig 1).

### Enhanced expression levels of DSB repair genes in aging cumulus cells

**Fig 2:**
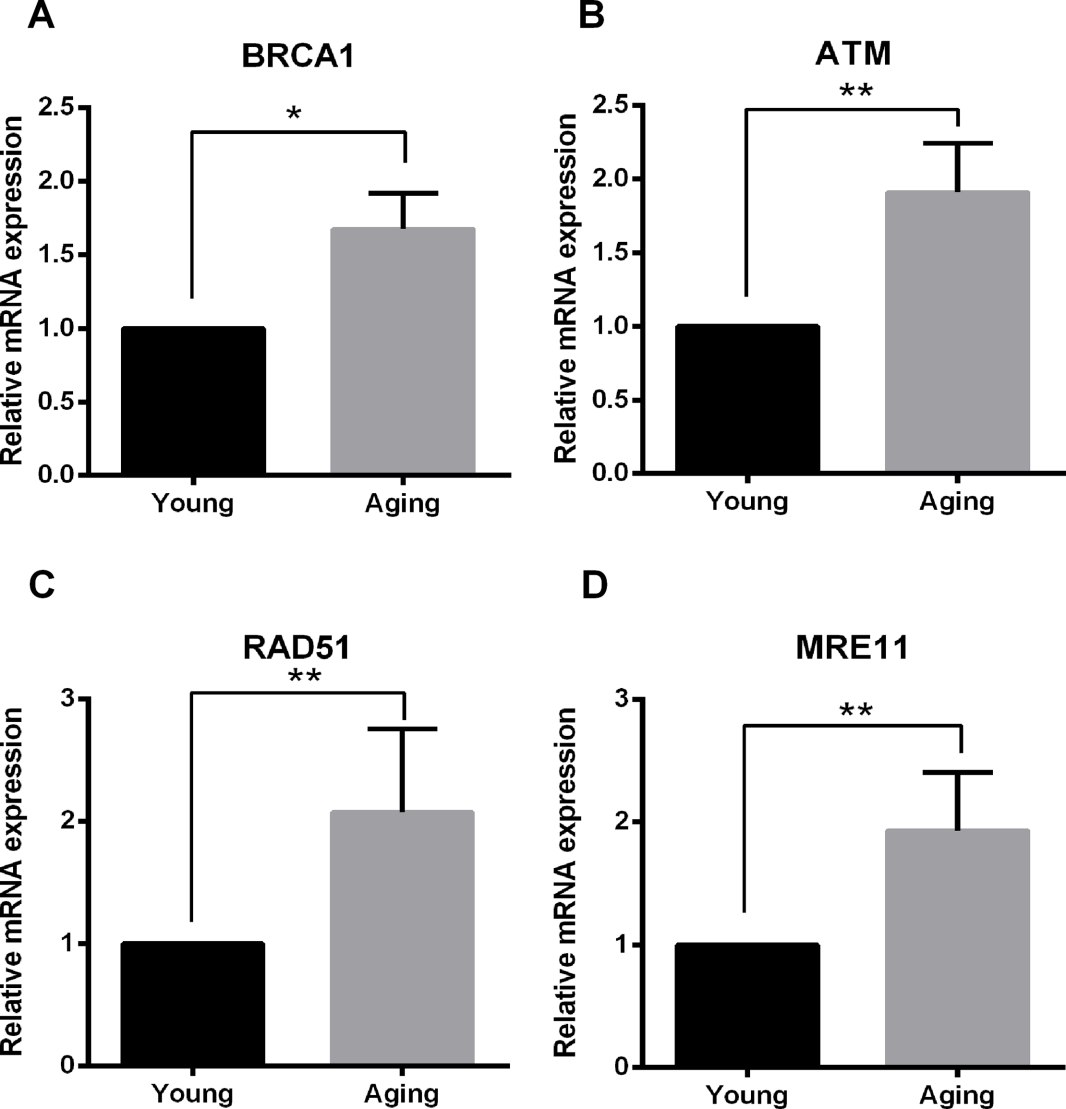
Relative expression levels of BRCA1 (A), ATM (B), RAD51 (C) and MRE11 (D) in young and aging cumulus cells. Results are reported as the mean ± SD from *n* = 5 independent experiments (significant difference, *P<0.05; **P<0.01).

Next, we sought to determine whether the expression of genes in the DSB repair pathway was activated in aging cumulus cells. As expected, BRCA1, ATM, meiotic recombination 11 homolog A (MRE11) and the RAD radiation repair gene (RAD51) showed higher expression levels in aging than in young cumulus cells (Fig 2).

### Increased protein levels of phosphorylation of H2AX and DSB repair genes in aging cumulus cell

**Fig 3:**
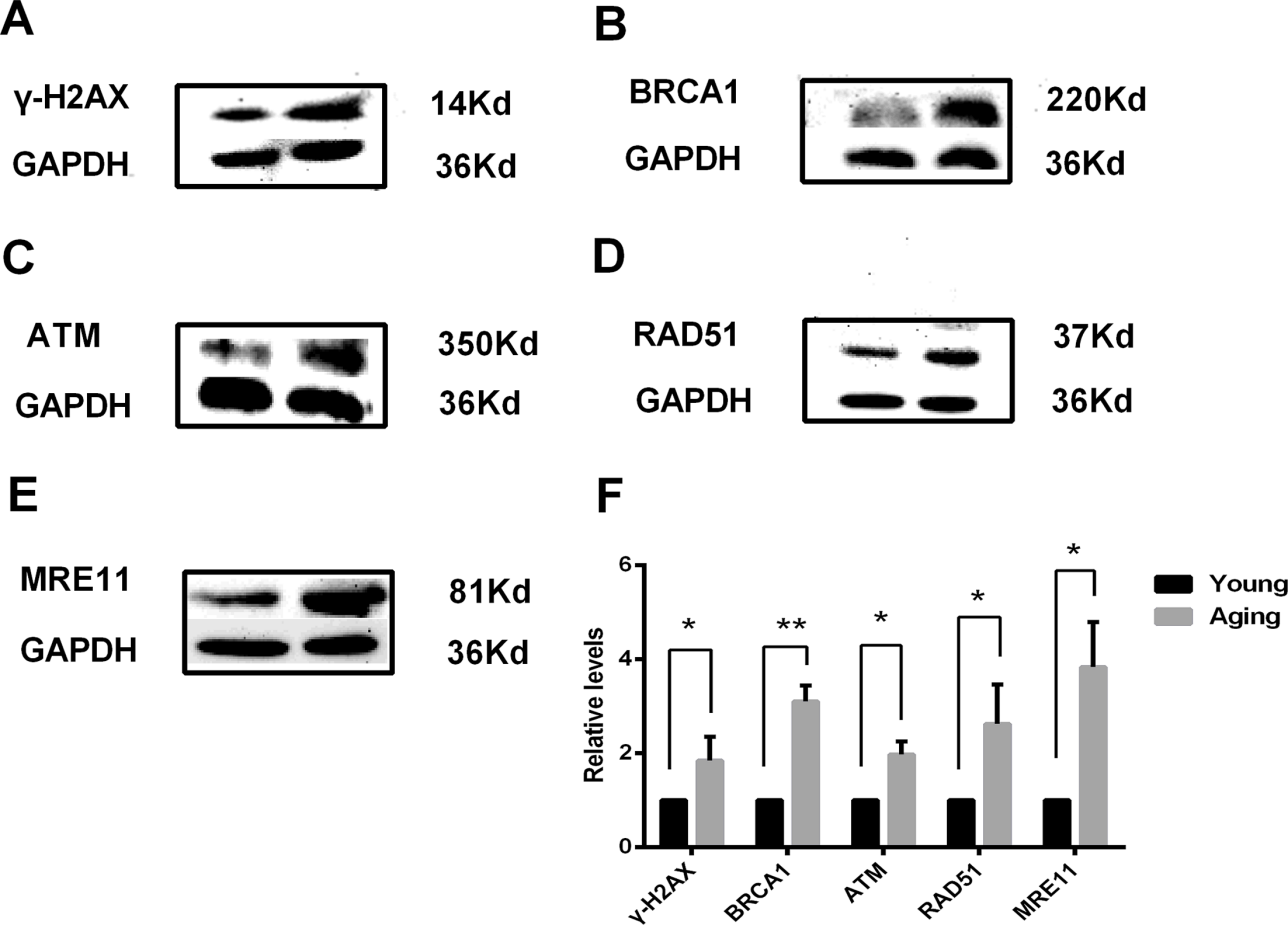
Western blot analyses of DSB repair proteins in young and aging cumulus cells (A, γ-H2AX; B, BRCA1; C ATM; D, RAD51; E, MRE11). Optical band density was calculated using ImageJ software and normalized based on GAPDH as an internal reference protein (F). The results are reported as the mean ± SD from *n* = 3 independent experiments (significant difference, *P<0.05; **P<0.01).

Western blot analysis of phosphorylated H2AX, BRCA1, ATM, MRE11 and RAD51 in aging and young cumulus cells was carried out using antibodies recognizing Ser-139 of γ-H2AX (~14 kDa), BRCA1 (~220 kDa), ATM(~350 kDa), MRE11(~81 kDa) and RAD51(~37 kDa), and the results were quantified based on signal intensity from three independent experiments. Consistent with the results of RT-qPCR, all tested genes also showed a significant increase in their protein levels (Fig 3).

### Increased apoptosis frequency in aging human cumulus cells

**Fig 4:**
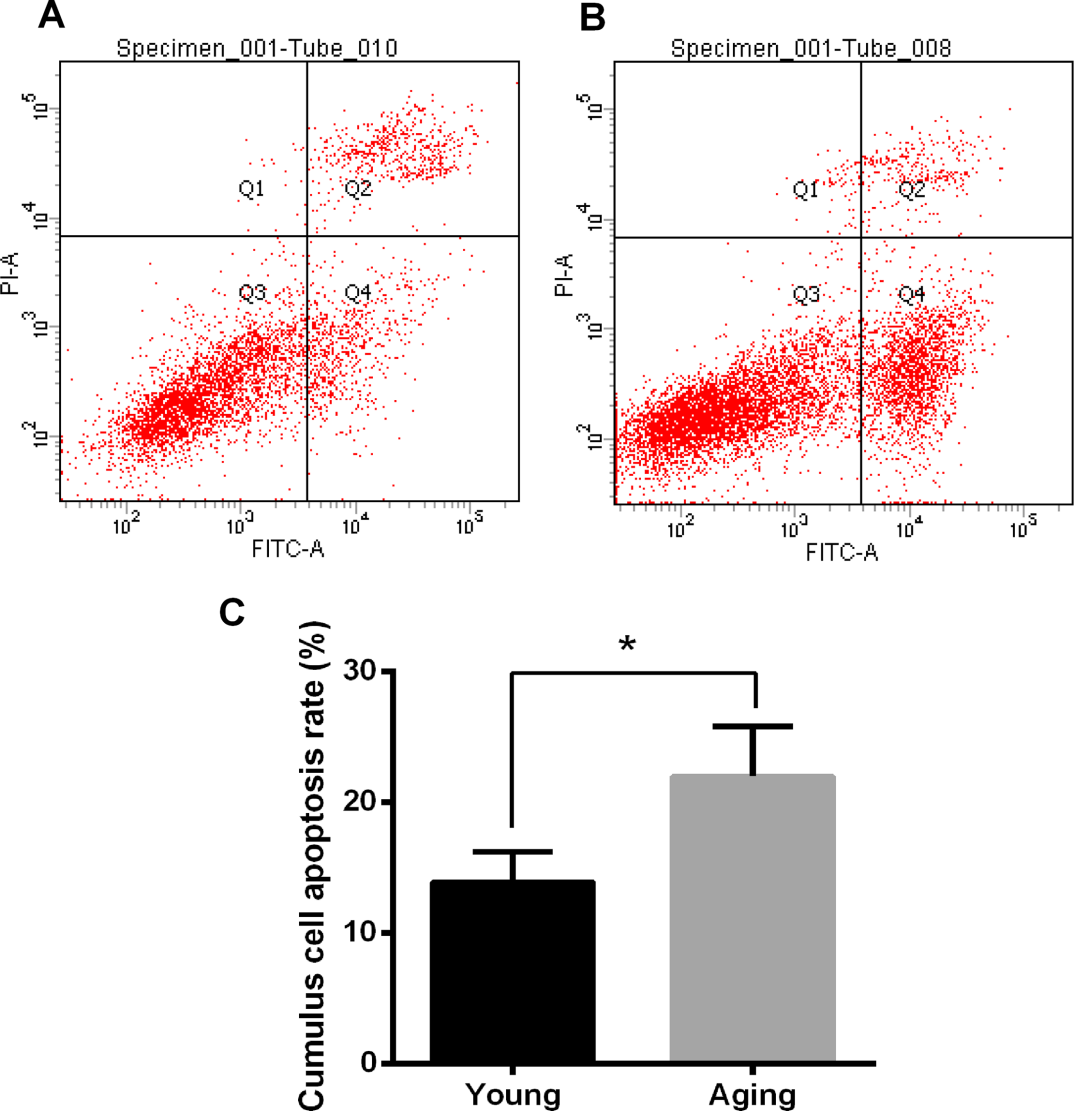
Detection of early apoptosis in aging and young cumulus cells. (A, Young; B, Aging) Representative images of raw flow cytometric data from young and aging cumulus cells obtained via staining with FITC Annexin-V. (C) Rates of apoptosis in the young and aging cumulus cells. The results are reported as the mean ± SD from *n* = 4 independent experiments (significant difference, *P<0.05).

Finally, we used flow cytometry to analysis the apoptosis in human cumulus cell by staining with FITC-Annexin-V/PI. Our results indicated that aging cumulus cells underwent the early stage of apoptosis, revealed by a significantly higher Annexin-V-positive rate (21.95±3.83% vs. 13.85±2.34%, P<0.05) (Fig 4).

## Discussion

Age is an established risk factor inducing reduced embryonic development and poor IVF-ET outcomes. Although poor oocyte quality is believed to be involved in the impaired developmental potential, the detailed mechanism remains largely unknown. A better understanding of this process will provide an improved reference for predicting, or even preventing, such complications. Our research provides the first evidence that significant DNA DSBs and, thus, the repair pathway activated in response exist in cumulus cells of aging women. Considering that unrepaired DSBs are always implicated in triggering apoptosis, our observations imply that DSBs in cumulus cells may be an important factor inducing oocyte impairment.

AMH is an important clinical indicator for the estimation of ovarian reserves and function [27]. It has been reported that patients with rather low AMH levels during controlled ovarian hyperstimulation produce reduced-quality COCs, as evaluated based on the rate of DSB-positive cumulus cells [28]. Previous studies have also shown that aged patients display decreased ovarian functions, together with lower serum AMH levels. Notably, aging cumulus cells showed an increased apoptosis rate and DNA fragmentation as well as a shortened telomere length [10].

In fact, many prior studies have focused on age-related aberrations in cumulus cells, including mitochondrial dysfunction due to oxidative stress [29–31]. Stress accumulation can further lead to DNA damage and telomere shortening, which adversely affect oocyte quality. However, the mechanism underlying this process remains largely unknown. To the best of our knowledge, our study is the first to associate the occurrence of apoptosis and DSBs in cumulus cells with abnormal hormone levels and poor subsequent embryonic development in aging IVF patients. Our study also supported the use of the DSB marker γ-H2AX as a non-invasive selection indicator with a clinical guidance value.

DSBs are identified by kinases and/or activated repair proteins. One of the most important members of this group is ATM, along with its homologous protein ATR, both of which are activated by DSBs [32]. Once activated, ATM is phosphorylated and recruited to DNA double-strand break sites and further activates phenomena such as cell apoptosis and cell cycle arrest [33–35]. The tumor suppressor protein BRCA1, a crucial member of the ATM-mediated DSB repair family, plays an important role in maintaining genetic integrity via interaction with a number of other proteins, including MRE11, RAD50 and BRCA2 [36]. BRCA1-deficient mice and women with germline BRCA1 mutations exhibit accelerated oocyte loss during aging [23].

It has generally been accepted that DNA damage is a major consequence of aging [37,38]. However, other authors believe that the cell lifespan depends on the effectiveness of DNA repair and consider aging to be the result of a decrease in DNA repair activity [39]. Studies in mice and humans have found that an impaired ATM-mediated DSB repair capacity in oocytes causes oocyte aging. In aged oocytes, the expression of DSB-related repair genes is decreased [23,24], including that of the gene encoding RAD51, whose recruitment and targeting at DSB sites are required for DSB repair [40]. The response of RAD51 expression in aging oocytes varies greatly among species [41].

In fact, as specialized somatic cells surrounding the oocytes, cumulus cells are susceptible to the follicular microenvironment. Many genes that are dysregulated in cumulus cells may contribute to aging [29,42,43]. It has been reported that angiogenic genes, including *ANGPTL4*, *LEPR*, *TGFBR3*, and *FGF2*, are overexpressed in cumulus cells from patients >37 years of age [31]. Zhang D *et al*. have shown that increases in the rate of DSBs and DNA repair inefficiency in rhesus monkey granulosa cells may be due to ovarian aging. Interestingly, although these authors found that DSBs increased, the expression of BRCA1 did not appear to be abolished with age, suggesting that granulosa cells in middle-aged monkeys maintain considerable DSB repair competence [22]. This finding is similar to that observed in the hematopoietic stem cells of aging patients [44]. Our data also showed that the expression of genes related to the ATM repair pathway was significantly increased in the aging cumulus cells of aging women.

It is important to understand the repair mechanisms that are closely linked with ovarian reserves. We observed an activated ATM-mediated DSB repair pathway in aging human cumulus cells during controlled superovulation. This finding is in accordance with the detection of early apoptosis, revealed by an increase in Annexin-V-positive cells. Our data may offer a new insight for further understanding the role of cumulus cells in mediating the poor reproductive performance of aging patients. Practically, the increases in DSBs and the activated repair pathway are potential indicators that may be used for predicting outcomes after IVF-ET treatment.

## Supporting information

S1 Table Raw data for Figs 1–4. (XLSX)
